# Cortical Layer‐Specific Remodelling of Parvalbumin and Perineuronal Net Networks in Alcohol Use Disorder

**DOI:** 10.1111/nan.70066

**Published:** 2026-02-04

**Authors:** Tamsin Karas, Asheeta A. Prasad

**Affiliations:** ^1^ Discipline of Neuroscience, School of Medical Sciences, Faculty of Medicine and Health University of Sydney Camperdown NSW Australia

**Keywords:** alcohol use disorder, cortex, human post‐mortem, parvalbumin, perineuronal nets

## Abstract

**Background:**

Alcohol use disorder (AUD) is a chronically relapsing condition marked by a pathological shift in behaviour, where excessive motivational drive predominates over cognitive control. Brodmann area 6 (BA6) is a key cortical region that integrates cognitive control with motor output and striatal circuits. Cellular alterations in the BA6 can shift from flexible, goal‐directed planning to habitual, compulsive behaviours.

**Method:**

Here, we examined cellular changes in the human post‐mortem cortex from AUD cases (*n* = 9) and age‐matched controls (*n* = 10). The density of parvalbumin (PV) neurons and perineuronal nets (PNN) was analysed from immunofluorescent‐stained sections. The number of PV neurons, PNN‐positive cells, and PV neurons colocalised with PNN across cortical layers II–VI in BA6 regions was quantified.

**Results:**

Across layers II/VI, the density of PV neurons and PNN was similar in the control and AUD groups, indicative of no cellular loss in the BA6. Analysis of the colocalisation of PV neurons with PNN revealed no effect in layers II/III (*p* = 0.720). However, there was a significant increase in colocalisation of PV neurons with PNN in layers IV (*p* = 0.043) and V/VI (*p* = 0.025) in the AUD compared to the control group.

**Conclusion:**

This study reveals layer‐specific remodelling of PNN and PV networks in the human cortex in AUD cases, suggesting shifts in AUD behaviours are potentially attributed to PV neuronal activity regulated by PNN in specific cortical layers. Together, this study identifies AUD‐related neuropathology and provides insight into the mechanisms underlying persistent alcohol‐seeking behaviour.

## Summary

- First evidence of cortical perineuronal net remodelling in human alcohol use disorder.
- Increased perineuronal net and parvalbumin colocalisation selectively in layers IV–VI in AUD.
- Suggests reduced plasticity in cortical output circuits driving habitual behaviour.

## Graphical abstract

Remodelling of perineuronal nets on cortical parvalbumin neurons in layers IV–VI occurs in human alcohol use disorder, suggests reduced plasticity in cortical output circuits driving habitual behaviour.

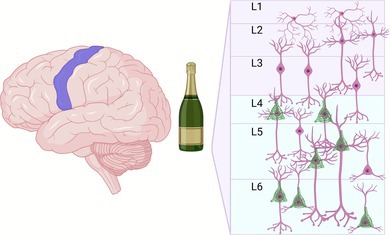

## 1 Introduction

Alcohol use disorder (AUD) is a chronically relapsing condition marked by compulsive alcohol‐seeking [1]. Compulsive behaviours in this neuropsychiatric disorder arise from neural network reorganisation, shifting control from goal‐directed to habitual responding through altered connectivity between motor‐related cortex and the dorsal striatum [2].

Changes in the cortex are most abundantly reported in AUD [3, 4]. Studies have identified reduced grey matter in the dorsolateral prefrontal cortex and diminished white matter integrity across cortical regions, with the frontal lobes particularly vulnerable [3, 5]. Notably, some research suggests that these volumetric reductions may not result from overt neuronal loss, but rather from structural changes such as dendritic retraction, soma shrinkage or cortical atrophy [6]. The cerebral cortex is a large area organised into functionally and histologically defined Brodmann areas. Brodmann Area 6 (BA6) is responsible for motor planning, sequencing and action selection [7].

Within the cerebral cortex, Brodmann areas are organised into six distinct layers (1–6), each with specialised cell types, patterns of connectivity and input–output relationships that collectively support complex cognitive and behavioural functions [5]. Layers 2 and 3 primarily contain small pyramidal neurons that facilitate local and long‐range communication between cortical regions. Layer 4, the internal granular layer, receives sensory input from the thalamus and is critical in processing environmental cues. Layer 5/6 contains large pyramidal neurons that constitute the cortex's principal output to regions such as the striatum, brainstem and spinal cord, structures involved in habit formation, motor control and reward processing [8]. The outputs of Layers 5/6 contribute to a feedback loop that correlates to the direct pathway of the basal ganglia and are also responsible for reward learning [9]. This anatomical relationship suggests these layers may contribute to the neural mechanisms underlying addiction.

Dispersed throughout cortical layers are parvalbumin‐positive (PV+) interneurons, fast‐spiking GABAergic cells that play a critical role in maintaining the excitatory and inhibitory balance within cortical circuits [10]. In rodent models of AUD, disruption of PV+ interneuron activity impairs performance on tasks requiring cognitive flexibility and executive control functions frequently compromised in addiction [11]. PV+ interneurons are often encapsulated by perineuronal nets (PNNs), which are specialised extracellular matrix structures that modulate neuronal excitability and stabilise synaptic connections [12]. PNNs are lattice‐like heterogeneous molecular structures of chondroitin sulphate proteoglycans (CSPG) and hyaluronans formed around neurons and glial cells within the extracellular matrix (ECM). PNNs regulate neuroplasticity by acting as structural barriers to plasticity, thereby reinforcing existing neural circuits [13, 14]. Increased expression of PNNs around PV neurons reduces plasticity and may drive maladaptive behaviour.

Several studies in AUD rodent models have reported increased PNN in the cortex, suggesting that PNNs hardwire neurons into relapse pathways, and disruption of PNNs can prevent relapse [12, 15, 16, 17]. Specifically, PNN density in the insular cortex but not in M1 or anterior cingulate cortex significantly increased following binge‐like alcohol intake, indicative of region‐specific remodelling in AUD [15]. In a separate study, using adolescent intermittent ethanol exposure, it was found that although the total number of PV+ interneurons remained stable, a greater percentage of these cells were surrounded by PNNs in the orbitofrontal and medial prefrontal cortices [17]. In addition to rodent findings, increases in PNNs have been documented in the hippocampus of subjects with substance use disorder and in nonhuman primates with chronic alcohol self‐administration [18]. Although these AUD rodent studies reveal a critical role of cortical PNNs in AUD, there is no evidence on human AUD, underscoring the need to explore remodelling in cortical regions. The present study aimed to address this gap by investigating changes in PV and PNN in AUD across cortical layers in BA6 using post‐mortem human brain tissue.

## 2 Methods

### 2.1 Human Post‐Mortem Brain Tissue

Formalin‐fixed and paraffin‐embedded 10‐μm coronal sections of post‐mortem human brain tissue from Brodmann area 6 (BA6) in the cortex were obtained from the NSW Brain Tissue Research Centre (BTRC). The BA6 region was collected from the prefrontal cortex block, superior frontal gyrus and in the coronal plane at the level just anterior to the anterior commissure. This research was conducted under approval from the University of Sydney Human Research Ethics Committee, project no. 2024/HE001120. A total of 20 cases were included, with 10 AUD and 10 controls. A case from the control group was excluded due to tissue damage. Clinical history per case was assessed by a psychiatric clinician to characterise and ensure cases met DSM‐IV criteria for AUD [19]. Control subjects were cleared of other neurodegenerative, neuropathological and neuropsychiatric diseases [1, 19, 20]. Case characteristics for the AUD sample group were matched with the control sample group for age, sex, and post‐mortem interval (PMI) (see Table 1).

**TABLE 1:** Demographic characteristics of control and AUD cases.

Group	Age	Sex	PMI	pH	RIN	Cause of Death	Period of alcohol use
Control (*n* = 9)	Patients with no neurological abnormalities or neuropathological disorders who consume less than 20 g of absolute alcohol per day						
Control	51	Male	37	7.00	7.9	Cardiac	33
Control	64	Male	19	6.55	7.2	Cardiac	44
Control	61	Male	31	6.69	7.5	Cardiac	46
Control	66	Male	39	6.66	7.6	Cardiac	48
Control	54	Male	31	6.36	5.4	Cardiac	35
Control	52	Male	31	6.28	6.4	Cardiac	0
Control	62	Male	32	6.67	6.5	Cardiac	37
Control	58	Male	70	6.82	6.9	Cardiac	32
Control	45	Female	33	6.78	7.6	Cardiac	20
AUD (*n* = 10)	‘Patients with no neurological abnormalities or neuropathological disorders who consume greater than 80 g per day’. Psychiatric clinician assessed all AUD cases to ensure cases met DSM‐V criteria for AUD.						
AUD	51	Male	32	6.80	7.8	Hepatic	36
AUD	62	Male	29	6.79	8.0	Cardiac/Respiratory	42
AUD	61	Male	40	6.63	5.2	Cardiac	35
AUD	82	Female	28	6.40	6.9	Hepatic	64
AUD	54	Male	36	6.43	8.4	Cardiac	38
AUD	51	Female	41	6.95	8.3	Toxicity	33
AUD	71	Male	43	6.80	7.1	Cardiac	53
AUD	56	Male	20	6.34	7.5	Hepatic/Blood loss	38
AUD	53	Male	79	6.79	7.7	Cardiovascular	38
AUD	32	Female	55	6.41	7.7	Toxicity	15

*Note:* Data presented for control (*n* = 9) and AUD (*n* = 10). Age is described years, post‐mortem interval (PMI) in hours, RNA integrity number (RIN), brain pH and years of drinking. NA = not applicable. There was no difference between groups for sex, age, PMI and brain pH.

### 2.2 Immunohistochemistry

Immunofluorescence staining was performed on paraffin‐embedded post‐mortem sections to detect PNNs and parvalbumin neurons. Sections were dewaxed in xylene and rehydrated through a graded series of ethanol baths (100%, 95%, 70% and 50%; 3 min each at room temperature). Next, antigen retrieval was conducted by heating sections in a citrate buffer (0.1 M, pH 6) using a steamer at 95°C–100°C (Breville, model BFS600) for 30 min. After retrieval, sections were rinsed in PBS and pretreated with a blocking buffer containing 10% bovine serum albumin for 1 h at room temperature. Blocked sections were incubated overnight at 4°C with primary antibodies, rabbit anti‐PV (1:500; Swant, Cat‐301, Switzerland) and mouse anti‐CSPG (1:600; MAB5284, Merk KGaA, Darmstadt, Germany), diluted in the blocking buffer. The next day, sections were washed in PBS containing 0.05% Tween‐20 and incubated in secondary antibodies, including donkey anti‐mouse Alexa Fluor 647 (1:1000, Invitrogen Cat. A31571) and goat anti‐rabbit Alexa Fluor 594 (1:1000; Invitrogen, Cat. A11012), diluted in blocking buffer. Sections were finally counterstained with DAPI and coverslipped using Prolong Diamond Antifade Mountant (Invitrogen, Cat. P36970).

### 2.3 Imaging, Region Identification and Quantification

**FIGURE 1:**
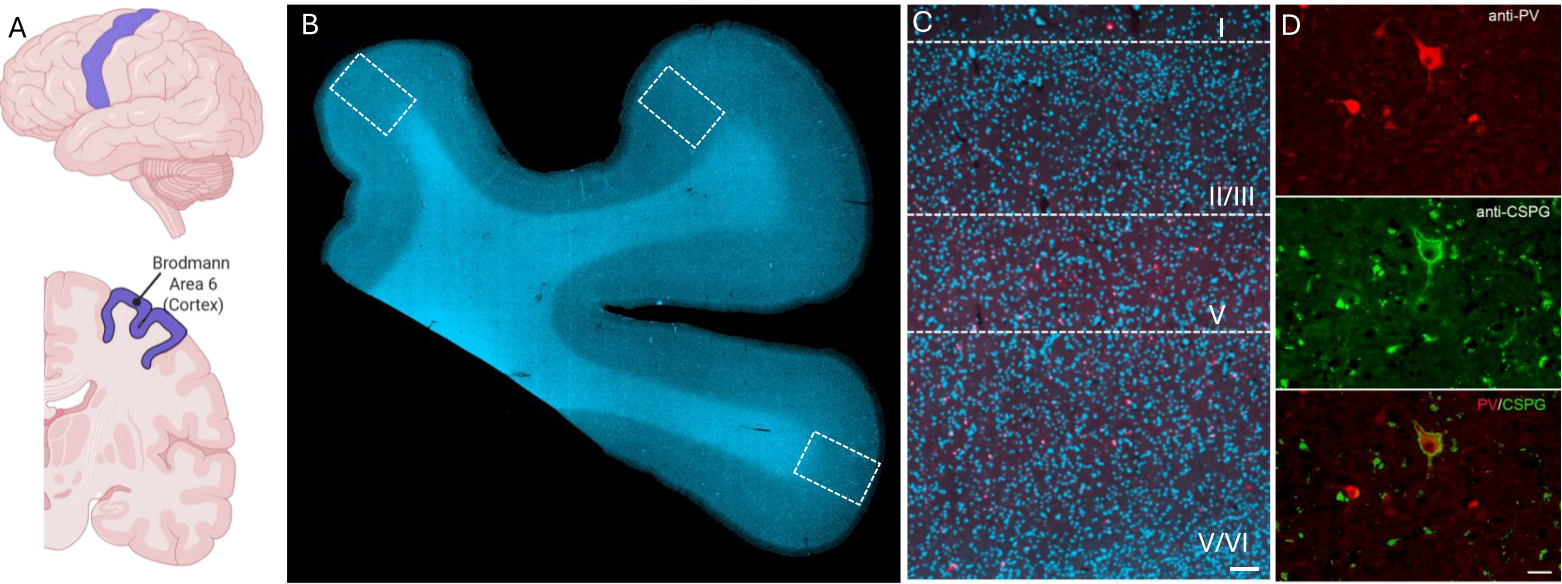
Expression of PNN and PV in the human BA6 region. (A) Anatomical localisation of Brodmann area 6 (B) Human post‐mortem brain sections from BA6 counterstained with DAPI (Blue) showing three regions of interest per brain section were analysed (C) Delineation used to quantify PVN/PNN density across layers I–VI, Scale bar = 50 μm (D) Representative immunofluorescent image of parvalbumin‐positive neuron (red) and perineuronal net (green). Scale bar = 5 μm.

Sections were visualised and scanned at 20x magnification using a Zeiss Axioscan 7 light microscope and analysed using QuPath‐0.5.1 software. On each sample, cortical Layers 1, 2/3, 4 and 5/6 were delineated on the gyri using anatomical boundaries identified using PV+ cell distribution. Three regions of interest (ROI) of 200 × 200 μm grid across the sample were outlined, covering the medial to lateral portion of the cortex. Immunoreactive PV and PNN cells were distinguished by labelled fluorophore, and morphology was confirmed through colocalisation with a nucleolus as revealed by DAPI staining. Colocalisation of PV+ and PNNs was based on the overlap of the two stains (see Figure 1A–D). All counts were normalised to the respective area analysed and expressed as cell density, calculated as the number of cells per μm^2^. All quantification was performed by an investigator blinded to case details. Images of the whole slide were captured, and a blind colocalisation of PNNs and PV in brain sections was performed manually using QuPath.

### 2.4 Statistical Analysis

Statistical analysis was conducted using IBM SPSS Statistics 26. Statistical significance was defined as *p* < 0.05, and results are reported as mean ± SEM for all analyses. Analyses were performed with a 95% confidence interval. Demographic analysis between groups was analysed using an independent samples *t*‐test. For comparison of PV and PNN across three layers, a one‐way ANOVA was used with a Bonferroni post hoc test. Normality was assessed for all datasets using the Shapiro–Wilk test. Data violating normality (*p* < 0.05) were analysed using the nonparametric Mann–Whitney *U* test.

## 3 Results

### 3.1 Demographic Analysis

Groups were matched for age, RNA integrity number (RIN), post‐mortem interval (PMI) and brain pH. Case demographics are summarised in Table 1. Independent *t*‐tests indicated no significant differences between the control and AUD groups, respectively, in age (57.00 ± 2.31, 57.30 ± 4.19), *F*_(1,17)_ = 1.30*, p* = 0.27; PMI (35.88 ± 4.64, 40.30 ± 5.27), *F*_(1,17)_ = 0.30*, p* = 0.58; RIN (7.00 ± 0.26, 7.460 ± 0.29), *F*_(1,17)_ = 0.00*, p* = 0.95; or pH (6.64 ± 0.07, 6.63 ± 0.69), *F*_(1,17)_ = 0.24*, p* = 0.62.

### 3.2 Quantification of PNN and PV Neurons Across BA6 Cortical Layers

**FIGURE 2:**
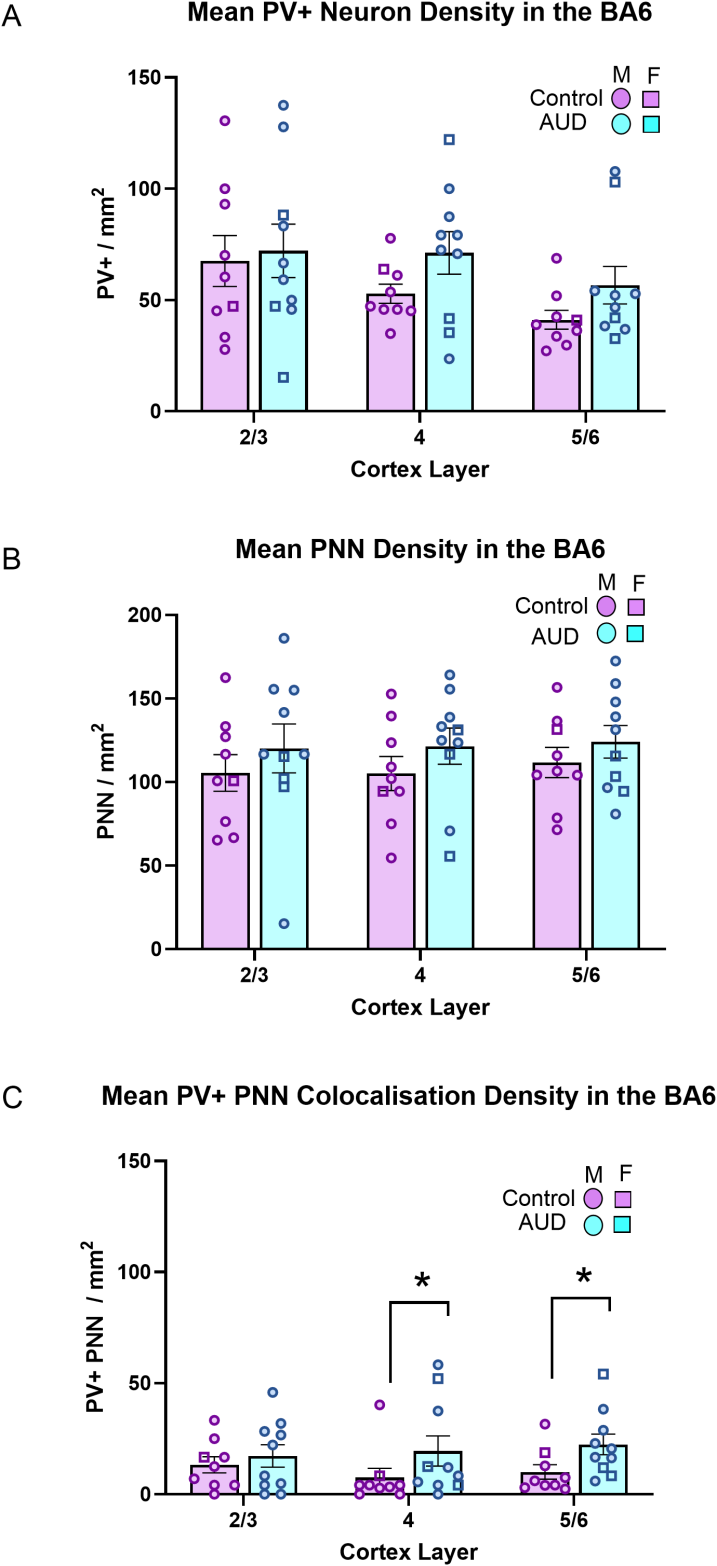
Quantification of PNN and PV neurons across cortical layers in BA6. PV+ cell, PNN and PV+/PNN colocalisation densities across cortical Layers 2/3, 4 and 5/6 in control and AUD cases in Brodmann area 6. Data are represented as circles, and those for females as squares. (A) No significant differences were observed within or between groups for PV+ density (B) or PNNs. (C) PV+/PNN colocalisation shows no significant differences were found in Layers 2/3; however, AUD cases showed higher densities in AUD compared to controls in Layers 4 and 5/6 (*p* < 0.05).

One‐way ANOVA analysis of PV density across three layers in the control group showed similar density: II/III PV (67.51 ± 11.42), IV PV (52.82 ± 4.28) and V/VI PV (53.82 ± 4.64) *F*_(2,24)_ = 3.14, *p* = 0.06. Similarly, the PNN density in the control group showed no difference across layers: II/III PNN (105.57 ± 10.95), IV PNN (105.06 ± 10.21) and V/VI PNN (111.72 ± 9.025), *F*_(2,24)_ = 0.13, *p* = 0.87. A comparison within the AUD cohort of PV density across layers also showed no difference: II/III PV (78.40 ± 11.24), IV PV (68.55 ± 10.75) and V/VI PV (57.54 ± 8.20); *F*_(2,26)_ = 1.04, *p* = 0.36. Analysis of PNN density in the AUD across layers showed no differences either: II/III PNN (122.15 ± 16.16), IV PNN (126.13 ± 8.35) and V/VI PNN (119.30 ± 11.76), *F*_(2,26)_ 0.08*, p* = 0.92. To assess if PMI impacted the number of PNNs or PV cells, Pearson correlation analysis for PNN and PV counts across all layers was statistically insignificant, *p* > 0.05, Figure 2A,B.

### 3.3 Comparison Between Groups in the PV and PNN Densities Across Layers Between Groups

Comparison of PNN densities across layers between the control and AUD groups showed no difference either; II/III PNN *F*_(1,17)_ = 0.19*, p* = 0.66, IV PNN *F*_(1,17)_ = 0.01*, p* = 0.91 and V/VI PNN *F*_(1,17)_ = 0.53*, p* = 0.46. Comparison of PV densities across layers between the control and AUD groups showed no difference either; II/III PV *F*_(1,17)_ = 0.05*, p* = 0.81, IV PV *F*_(1,17)_ = 3.65*, p* = 0.07 and V/VI PV *F*_(1,17)_ = 2.84*, p* = 0.11.

### 3.4 Quantification of PNN and PV Colocalization Across Cortical Layers in BA6

The Mann–Whitney *U* test was used to analyse PV colocalisation with PNN between groups and layers. No group difference was detected in layers II/III (*U* = 50.00, *p* = 0.72); however, there was a significant difference for layer IV (*U* = 70.00, *p* = 0.04) and layer V/VI (*U* = 72.00, *p* = 0.02), Figure 2C.

## 4 Discussion

PNNs have been previously described in BA6 with co‐localisation with PV neurons and astrocytes [21]. Here, we examined both PV neurons and PNN in the BA6 area in control and AUD cases. Overall, our findings align with previous studies with similar levels of PV and perineuronal nets in the human cortex and extend to examine changes in AUD [21, 22]. We report no change in the density of PNN or PV neurons in the AUD group compared to controls; however, we identified a layer‐specific shift in PNN colocalization with PV neurons. As hypothesised based on findings from preclinical AUD models that reported significant changes in PNN and PV neurons, changes in human AUD cases were anticipated [12, 14, 15, 16, 23]. For the first time, we report PNN changes in the human AUD cortex, with increased localisation of PNNs on PV neurons, specifically in layers V–VI.

Our findings show no significant differences in PV+ alone or in PNN density across cortical layers or between groups, indicating that AUD either does not lead to neuronal cell loss in BA6 or is limited to the white matter. Similar to previous volumetric studies, suggesting the loss of volume in the cortex is predominantly in the white matter [3].

### 4.1 Layer‐Specific Changes

This specific alteration in layers V‐VI sheds light on the role of specific cortical inputs and outputs in AUD. Layers V‐VI are primarily involved in cortical output to regions such as the thalamus and striatum, key components of the basal ganglia's direct pathway. This may reflect diminished plasticity within circuits associated with action selection behaviour. The increase in PNNs around PV+ interneurons suggests a reduction in neural plasticity leading to maladaptive stabilisation of alcohol‐seeking habits, reinforcing compulsive behaviours and perpetuating the cycle of addiction. Our findings of layer‐specific remodelling of PNN and PV networks in the human cortex resonate with previously reported extracellular matrix adaptations in the hippocampus of individuals with substance use disorder [18]. Layer‐specific alterations have been reported in the cortex of other neuropsychiatric disorders [24]. Based on the layer‐specific effect in AUD, future PET or FMRI studies should examine layer resolution.

### 4.2 Limitations of Study

Our study included 15 males and only four females across both groups, limiting the capacity to detect sex‐specific effects. A more gender‐balanced sample is also necessary for meaningful analysis of sex‐related differences. The current study investigating gender differences is particularly important, as it may have significant implications for the development of targeted treatments for individuals with AUD. For instance, Martins de Carvalho et al. reported that female mice displayed greater PNN staining intensity in the insular cortex during aversion‐resistant drinking than males, suggesting a heightened PNN responsiveness in females. In contrast, Towner et al. found increased PNN expression in males but not females following AIE exposure [16, 25]. Furthermore, changes in oestrogen levels during the reproductive cycle in female rats have been shown to impact PNN development [26]. Such findings imply that PNN dynamics may vary by sex, potentially influencing vulnerability to or the progression of AUD.

### 4.3 Human and Rodent Models: Validity and Future Mechanistic Insights

These findings align with a previous rodent study, which found an increased colocalisation of PNNs with PV+ cells in other cortical regions of the orbital and medial prefrontal cortex of male rats exposed to alcohol [16]. Although the study did not examine laminar‐specific differences, the results support the interpretation that interactions between PNNs and PV cells may contribute to alcohol‐related impairments in cortical plasticity. This study underscores the translational relevance of AUD animal models, highlighting their utility in unravelling the cellular mechanisms underlying AUD. The conserved reward system across vertebrates further supports the applicability of these models in the mechanistic study and manipulation of pathology in AUD. Although the findings reported here show neural changes in AUD cases, we cannot eliminate whether these changes are induced by alcohol exposure alone or contribute to relapse to alcohol‐seeking behaviour. Manipulation of PNN in experimental approaches is limited to degradation of PNNs using chondroitinase ABC (ChABC). ChABC has been successfully applied in rodent addiction models to prevent morphine‐induced and cocaine‐induced reinstatement [14, 27]. However, in our study, rather than a loss or gain of PNN, we identified a shift in the localisation of PNN on PV neurons. A functional demonstration of our finding would require specifically targeting PNN on PV neurons, limiting the capacity to address this issue. Thus, although a phenotypic change was observed, we cannot conclude its functional consequences.

### Conclusions

This study translates the growing body of preclinical literature implicating perineuronal nets in the pathophysiology of AUD. We found no difference in the density of PV+ neurons and PNNs between sample groups, indicating that AUD may not cause neuronal cell loss. The key finding is a significant increase in the co‐localisation of PV+ interneurons with PNNs in layers 4–6 of BA6 in AUD, which indicates reduced plasticity in cortical output circuits that contribute to habit formation. This laminar‐specific change may contribute to the persistence of compulsive alcohol‐seeking behaviours in AUD by reinforcing maladaptive neural patterns. Overall, our findings provide insight into neurological changes that may underlie the behavioural shift in alcohol addiction.

## Author Contributions

T.K. carried out all quantification and analysis of data. A.A.P. conceived, designed and prepared the manuscript.

## Ethics Statement

This research was conducted under approval from the University of Sydney Human Research Ethics Committee, project no. 2024/HE001120).

## Conflicts of Interest

The authors declare no conflicts of interest.

## Data Availability

The data that support the findings of this study are available from the corresponding author upon reasonable request.

## References

[1] A. E. Rasool , C. Peat , J. Liu , G. Sutherland , and A. A. Prasad , “Post‐Mortem Human Brain Analysis of the Ventral Pallidum in Alcohol Use Disorder,” Addiction Neuroscience 13 (2024): 100180.

[2] G. F. Koob and N. D. Volkow , “Neurocircuitry of Addiction,” Neuropsychopharmacology 35, no. 1 (2010): 217–238. 19710631 10.1038/npp.2009.110 PMC2805560

[3] S. M. de la Monte and J. J. Kril , “Human Alcohol‐Related Neuropathology,” Acta Neuropathologica 127, no. 1 (2014): 71–90. 24370929 10.1007/s00401-013-1233-3 PMC4532397

[4] A. E. Rasool , J. L. Cornish , and A. A. Prasad , “The Neuropathology of Alcohol Use Disorder: Cellular Insights From Human Post‐Mortem Studies,” Journal of Neurochemistry 169, no. 9 (2025): e70233. 40931957 10.1111/jnc.70233 PMC12424107

[5] K. Abernathy , L. J. Chandler , and J. J. Woodward , “Alcohol and the Prefrontal Cortex,” International Review of Neurobiology 91 (2010): 289–320. 20813246 10.1016/S0074-7742(10)91009-X PMC3593065

[6] J. J. Kril , G. M. Halliday , M. D. Svoboda , and H. Cartwright , “The Cerebral Cortex Is Damaged in Chronic Alcoholics,” Neuroscience 79, no. 4 (1997): 983–998. 9219961 10.1016/s0306-4522(97)00083-3

[7] S. Tanaka , M. Honda , and N. Sadato , “Modality‐Specific Cognitive Function of Medial and Lateral Human Brodmann Area 6,” Journal of Neuroscience 25, no. 2 (2005): 496–501. 15647494 10.1523/JNEUROSCI.4324-04.2005 PMC6725497

[8] Y. Miyashita , “Operating Principles of the Cerebral Cortex as a Six‐Layered Network in Primates: Beyond the Classic Canonical Circuit Model,” Proceedings of the Japan Academy. Series B, Physical and Biological Sciences 98, no. 3 (2022): 93–111. 35283409 10.2183/pjab.98.007 PMC8948418

[9] P. Calabresi , B. Picconi , A. Tozzi , V. Ghiglieri , and M. di Filippo , “Direct and Indirect Pathways of Basal Ganglia: A Critical Reappraisal,” Nature Neuroscience 17, no. 8 (2014): 1022–1030. 25065439 10.1038/nn.3743

[10] Q. Zhu , W. Ke , Q. He , et al., “Laminar Distribution of Neurochemically‐Identified Interneurons and Cellular Co‐Expression of Molecular Markers in Epileptic Human Cortex,” Neuroscience Bulletin 34, no. 6 (2018): 992–1006. 30171525 10.1007/s12264-018-0275-x PMC6246828

[11] A. J. Murray , M. U. Woloszynowska‐Fraser , L. Ansel‐Bollepalli , et al., “Parvalbumin‐Positive Interneurons of the Prefrontal Cortex Support Working Memory and Cognitive Flexibility,” Scientific Reports 5 (2015): 16778. 26608841 10.1038/srep16778 PMC4660359

[12] H. Chen and A. W. Lasek , “Perineuronal Nets in the Insula Regulate Aversion‐Resistant Alcohol Drinking,” Addiction Biology 25, no. 6 (2020): e12821. 31433552 10.1111/adb.12821 PMC7032993

[13] M. Slaker , J. Barnes , B. A. Sorg , and J. W. Grimm , “Impact of Environmental Enrichment on Perineuronal Nets in the Prefrontal Cortex following Early and Late Abstinence From Sucrose Self‐Administration in Rats,” PLoS ONE 11, no. 12 (2016): e0168256. 27977779 10.1371/journal.pone.0168256 PMC5158028

[14] M. Slaker , L. Churchill , R. P. Todd , et al., “Removal of Perineuronal Nets in the Medial Prefrontal Cortex Impairs the Acquisition and Reconsolidation of a Cocaine‐Induced Conditioned Place Preference Memory,” Journal of Neuroscience 35, no. 10 (2015): 4190–4202. 25762666 10.1523/JNEUROSCI.3592-14.2015 PMC4355195

[15] H. Chen , D. He , and A. W. Lasek , “Repeated Binge Drinking Increases Perineuronal Nets in the Insular Cortex,” Alcoholism, Clinical and Experimental Research 39, no. 10 (2015): 1930–1938. 26332441 10.1111/acer.12847 PMC4592458

[16] T. T. Towner , H. J. Coleman , M. A. Goyden , et al., “Prelimbic Cortex Perineuronal Net Expression and Social Behavior: Impact of Adolescent Intermittent Ethanol Exposure,” Neuropharmacology 262 (2025): 110195. 39437849 10.1016/j.neuropharm.2024.110195 PMC12522164

[17] C. A. Dannenhoffer , A. Gómez‐A , V. A. Macht , et al., “Impact of Adolescent Intermittent Ethanol Exposure on Interneurons and Their Surrounding Perineuronal Nets in Adulthood,” Alcoholism, Clinical and Experimental Research 46, no. 5 (2022): 759–769. 35307830 10.1111/acer.14810 PMC9117471

[18] J. Valeri , C. Stiplosek , S. M. O'Donovan , et al., “Extracellular Matrix Abnormalities in the Hippocampus of Subjects With Substance Use Disorder,” Translational Psychiatry 14, no. 1 (2024): 115. 38402197 10.1038/s41398-024-02833-y PMC10894211

[19] A. E. Rasool , T. Furlong , and A. A. Prasad , “Microglia Activity in the Human Basal Ganglia Is Altered in Alcohol Use Disorder and Reversed With Remission From Alcohol,” Addiction Biology 29, no. 2 (2024): e13374. 38380734 10.1111/adb.13374 PMC10898843

[20] C. Harper , G. Dixon , D. Sheedy , and T. Garrick , “Neuropathological Alterations in Alcoholic Brains. Studies Arising From the New South Wales Tissue Resource Centre,” Progress in Neuro‐Psychopharmacology & Biological Psychiatry 27, no. 6 (2003): 951–961. 14499312 10.1016/S0278-5846(03)00155-6

[21] D. Virgintino , D. Perissinotto , F. Girolamo , et al., “Differential Distribution of Aggrecan Isoforms in Perineuronal Nets of the Human Cerebral Cortex,” Journal of Cellular and Molecular Medicine 13, no. 9b (2009): 3151–3173. 19220578 10.1111/j.1582-4934.2009.00694.x PMC4516474

[22] I. Banovac , M. V. Prkačin , I. Kirchbaum , et al., “Morphological and Molecular Characteristics of Perineuronal Nets in the Human Prefrontal Cortex—A Possible Link to Microcircuitry Specialization,” Molecular Neurobiology 62, no. 1 (2025): 1094–1111. 38958887 10.1007/s12035-024-04306-1 PMC11711633

[23] Y. X. Xue , L. F. Xue , J. F. Liu , et al., “Depletion of Perineuronal Nets in the Amygdala to Enhance the Erasure of Drug Memories,” Journal of Neuroscience 34, no. 19 (2014): 6647–6658. 24806690 10.1523/JNEUROSCI.5390-13.2014 PMC6608143

[24] N. Y. Larsen , N. Vihrs , J. Møller , et al., “Layer III Pyramidal Cells in the Prefrontal Cortex Reveal Morphological Changes in Subjects With Depression, Schizophrenia, and Suicide,” Translational Psychiatry 12, no. 1 (2022): 363. 36064829 10.1038/s41398-022-02128-0 PMC9445178

[25] L. Martins de Carvalho , H. Chen , M. Sutter , and A. W. Lasek , “Sexually Dimorphic Role for Insular Perineuronal Nets in Aversion‐Resistant Alcohol Consumption,” Front Psychiatry 14 (2023): 1122423. 36926460 10.3389/fpsyt.2023.1122423 PMC10011443

[26] N. Uriarte , M. Ferreño , D. Méndez , and J. Nogueira , “Reorganization of Perineuronal Nets in the Medial Preoptic Area During the Reproductive Cycle in Female Rats,” Scientific Reports 10, no. 1 (2020): 5479. 32214157 10.1038/s41598-020-62163-z PMC7096482

[27] Y.‐X. Xue , Y. X. Luo , P. Wu , et al., “A Memory Retrieval‐Extinction Procedure to Prevent Drug Craving and Relapse,” Science 336, no. 6078 (2012): 241–245. 22499948 10.1126/science.1215070 PMC3695463

